# Maternal deaths in Sagamu in the new millennium: a facility-based retrospective analysis

**DOI:** 10.1186/1471-2393-6-6

**Published:** 2006-03-10

**Authors:** Olufemi T Oladapo, Mustafa A Lamina, Tuminu A Fakoya

**Affiliations:** 1 Maternal and Fetal Health Research Unit, Department of Obstetrics and Gynaecology, Obafemi Awolowo College of Health Sciences/Olabisi Onabanjo University Teaching Hospital, Sagamu, Ogun State, Nigeria

## Abstract

**Background:**

Health institutions need to contribute their quota towards the achievement of the Millennium Development Goal (MDG) with respect to maternal health. In order to do so, current data on maternal mortality is essential for careproviders and policy makers to appreciate the burden of the problem and understand how best to distribute resources. This study presents the magnitude and distribution of causes of maternal deaths at the beginning of the 21st century in a Nigerian referral hospital and derives recommendations to reduce its frequency.

**Methods:**

A retrospective descriptive analysis of all cases of maternal deaths at Olabisi Onabanjo University Teaching Hospital, Sagamu, Southwest Nigeria between 1 January 2000 to 30 June 2005.

**Results:**

There were 75 maternal deaths, 2509 live births and 2728 deliveries during the study period. Sixty-three (84.0%) of the deaths were direct maternal deaths while 12 (16.0%) were indirect maternal deaths. Major causes of deaths were hypertensive disorders in pregnancy (28.0%), haemorrhage (21.3%) and sepsis (20.0%). Overall, eclampsia was the leading cause of deaths singly accounting for 24.0% of all maternal deaths. Abortion and HIV-related mortality accounted for 1.3% and 4.0% of maternal deaths, respectively. The maternal mortality ratio of 2989.2 per 100,000 live births was significantly higher than that reported for 1988–1997 in the same institution. Up to 67/794 (8.4%) patients referred from other facilities died compared to 8/1934 (0.4%) booked patients (OR: 22.1; 95% CI: 10.2–50.1). Maternal death was more likely to follow operative deliveries than non-operative deliveries (27/545 vs 22/2161; OR: 5.07; 95% CI: 2.77–9.31).

**Conclusion:**

At the middle of the first decade of the new millennium, a large number of pregnant women receiving care in this centre continue to die from preventable causes of maternal death. Adoption of evidence-based protocol for the management of eclampsia and improvement in the quality of obstetric care for unbooked emergencies would go a long way to significantly reduce the frequency of maternal deaths in this institution.

## Background

The last decade of the 20th century witnessed intensive mobilisation of international health and development agencies to reduce maternal deaths in the developing countries. Yet, there was no significant reduction in the maternal mortality ratio (MMR) in Nigeria. The failure of the Safe Motherhood Initiative, proposed by the World Health Organization (WHO) in 1987, and other similar programmes in globally addressing the issue of maternal mortality by the year 2000 led to setting of new goals for the present millennium. One of the key Millennium Development Goals (MDG), which the United Nations Member States pledged to meet by 2015, is improvement in maternal health by reduction in the MMR to one-quarter of the 1990 data [1]. Among the principal obstacles to appropriate distribution of resources targeted towards improving maternal healthcare is the lack of accurate data on the number, causes and local factors influencing adverse maternal outcomes. In spite of the large number of maternal deaths, national statistics in most parts of sub-Saharan Africa are either not available or unreliable. This problem is partly attributable to the limitations of the methods for population-based measurement of maternal mortality, which cannot be readily overcome by low resource countries due to numerous socio-economic, infrastructural and cultural barriers [2,3]. Consequently, majority of the maternal mortality data from these settings are obtained from hospital-based studies. In spite of their shortcomings, hospital-based studies are relatively easy to perform and can also provide substantial and useful information, even if the results are likely to be influenced by referral bias [4,5]. Local enquiries into maternal deaths over a given period can be used to monitor as well as indicate measures for improving the quality of obstetric care provided in a health facility [5].

In order for health institutions to contribute their quota towards the achievement of the MDG with respect to maternal health, current data on maternal mortality is essential for their careproviders and policy makers to appreciate the burden of the problem and understand how best to distribute resources. This study presents the magnitude and distribution of causes of maternal mortality at the beginning of the 21st century in a Nigerian referral hospital and derives recommendations, to reduce the frequency of maternal deaths in this centre and in other centres in similar setting.

## Methods

### Hospital setting and study design

The study retrospectively reviewed all the maternal deaths that were recorded at Olabisi Onabanjo University Teaching Hospital (OOUTH), Sagamu Nigeria between 1 January 2000 and 30 June 2005. This hospital is funded by the government of Ogun State, in Southwest Nigeria and it serves as the referral centre for other health facilities in all the Local Government Areas in Remo and Ijebu regions of the State. These regions have a projected population of 1,186,282 people. In addition to providing emergency obstetric services to women referred from other centres, the hospital also provides antenatal care and delivery services for both unreferred low and high-risk pregnant women from Sagamu community and neighbouring towns. Patients are expected to pay for their services though in emergency situations, they are managed within the means of existing resources before funds are made available. The hospital provides blood transfusion services from limited stock and relatives of patients are requested to donate or pay for blood when blood transfusion is indicated, at times in cases of emergency.

### Definition of terms

In this study, maternal death was defined according to the tenth revision of International Classification of Diseases (ICD-10) by WHO [6]. It is described as the death of a woman while pregnant or within 42 days of termination of pregnancy, irrespective of the duration and the site of the pregnancy, from any cause related to or aggravated by the pregnancy or its management, but not from accidental or incidental causes. ***Direct ***maternal deaths are those resulting from complications of the pregnant state (pregnancy, labour and puerperium), from interventions, omissions, incorrect treatment, or from a chain of events arising from any of the above while ***indirect ***maternal deaths are those due to previously existing disease or disease that develop during pregnancy, and not due to direct obstetric causes but which was aggravated by the physiological effects of pregnancy. Maternal mortality ratio was defined as the number of maternal deaths per 100,000 live births. An "unbooked patient" in this study refers to a woman who did not utilise the antenatal care services of OOUTH, Sagamu.

### Data collection and analysis

Information was obtained from a combination of admission and discharge registers, labour and delivery records and retrieved case files from the Medical Records Department of the hospital. For each case of maternal death, data were collected on age, parity (during index pregnancy), booking status, mode of delivery and the cause of death. As the patients' relatives rarely allow autopsy, the causes of death were allocated to the consensus diagnoses arrived at during the monthly maternal mortality audit meetings, which were documented in the patients' case files. The total number of deliveries and live births conducted during the period of study were also documented. The Scientific and Ethical Committee of the Obstetrics and Gynaecology Department of OOUTH, Sagamu approved the study.

Data were entered into a computer database using Microsoft Excel software and analysed with Epi Info 2002 statistical package [7]. Results are presented in frequencies, percentages and summary statistics. MMR was determined for different age and parity groups and for each year of study. The confidence limits of the MMR for each year of study were also calculated at 95% confidence level. The relationship between maternal death, antenatal booking status and operative deliveries (caesarean section, instrumental delivery, destructive operations) was explored. The overall MMR for the reviewed period was compared with the figure reported for years 1988–1997 [8] in the same institution to note any change in the frequency of maternal death over time. Comparison of categorical variables was by computing the odds ratio (OR) at 95% confidence limits. Observed differences between two samples were considered statistically significant where p < 0.05 or confidence limits did not embrace unity (1)

## Results

During the period of study, there were 2728 deliveries, 2509 live births and 75 maternal deaths. Sixty-three (84.0%) of the deaths were direct maternal deaths while 12 (16.0%) were indirect maternal deaths. Table 1 shows the frequency of maternal deaths according to age and parity distribution of the women who died. The age ranged from 18 to 45 years with a mean of 28.9 ± 6.2 years. Over half of the maternal deaths occurred in women aged 20–29 years while about a quarter of them were 35 years and older. Approximately one-third of the women were experiencing their first childbirth. The MMR was lowest for those aged 25–29 years and highest for those aged ≥35 years. The noted differences were found to be statistically significant (χ^2 ^= 17.5, 4df; p = 0.0015). For the parity groups, the MMR was lowest in the Para 1–4 and highest in Para ≥5. Comparison of the MMRs for the parity groups did not show any statistically significant difference (Para 0 vs Para 1–4: OR: 1.32, CI: 0.77–2.24, p = 0.2806; Para 1–4 vs Para ≥5: OR: 0.42, CI: 0.17–1.25, p = 0.0583; Para 0 vs Para ≥5: OR: 0.56, CI: 0.22–1.71, p = 0.2531).

**Table 1:** Maternal deaths according to age and parity distribution

**Age (years)**	**Live births**	**Maternal deaths**	**MMR**
<20	72	4 (5.3)	5555.5
20–24	626	18 (24.0)	2875.4
25–29	979	21 (28.0)	2145.0
30–34	565	14 (18.7)	2477.9
≥35	267	18 (24.0)	6741.6
**Parity**			
0	698	24 (32.0)	3438.4
1–4	1711	45 (60.0)	2630.0
≥5	100	6 (8.0)	6000.0

MMR: Maternal mortality ratio

Percentages in parentheses

Sixty-seven (8.4%) of the maternal deaths were recorded among the 794 women who were not booked for antenatal care and delivery services at OOUTH, Sagamu while eight (0.4%) of 1934 booked patients died. Therefore, the risk of maternal deaths among unbooked patients was about 22 times that of booked patients (OR: 22.1; 95% CI: 10.2–50.1; p < 0.0001). Thirty-nine (58.2%) of the unbooked patients who died received antenatal care and attempted to deliver at either a Primary Healthcare Centre or General (district) hospital from where they were referred.

Table 2 shows the various causes of maternal deaths during the period of study. In descending order of frequency, the major categories were hypertensive disorders in pregnancy (28.0%), haemorrhage (21.3%) and sepsis (20.0%). Overall, eclampsia was the leading cause of deaths singly accounting for almost a quarter of all maternal deaths. Most (10/16) of the deaths due to haemorrhage were cases of postpartum haemorrhage. There was no death due to placenta praevia and abortion-related haemorrhage. A woman died from intraperitoneal haemorrhage caused by an avulsed subserous fibroid during pregnancy. Two (4.0%) of the maternal deaths resulted from complications of HIV infection, which became worse during the course of delivery and immediate puerperium. Pulmonary embolism was responsible for only 1.3% of maternal deaths.

**Table 2:** Causes of maternal deaths in Sagamu, Nigeria

**Cause**	**Maternal deaths (n)**	**Percentage**
**Haemorrhage**	**16**	**21.3**
*Early pregnancy*		
Ectopic pregnancy	2	2.7
Abortion	-	-
*Late pregnancy*		
Placenta praevia	-	-
Abruptio placentae	3	4.0
Postpartum haemorrhage	10	13.3
Others	1	1.3
**Hypertensive disorders**	**21**	**28.0**
Eclampsia	18	24.0
Severe pre-eclampsia	3	4.0
**Dystocia**	**7**	**9.3**
Uterine rupture	6	8.0
Obstructed labour	1	1.3
**Sepsis**	**15**	**20.0**
Puerperal sepsis	4	5.3
Intrapartum chorioamnionitis	10	13.3
Septic abortion	1	1.3
**Pulmonary embolism**	**1**	**1.3**
**Anaesthetic complication**	**1**	**1.3**
**Medical disorders**	**12**	**16.0**
Sickle cell crisis	2	2.7
Hypertensive/congestive cardiac failure	2	2.7
Anaemia (not due to haemorrhage	6	8.0
HIV infection	2	4.0

Among the 71 patients whose pregnancies reached the age of viability (≥28 weeks), 49 (69.0%) delivered before their demise while 22 (31.0%) died undelivered. Causes of death among women who died before delivery were cerebrovascular accident due to eclampsia (n = 8), acute renal failure following severe pre-eclampsia (n = 1), intrapartum gram-negative septisaemic shock (n = 7), massive haemorrhage from placenta abruption (n = 1), hypovolaemic shock due to uterine rupture (n = 1), sequestration crisis in sickle cell anaemia (n = 1) and cardiac failure secondary to severe anaemia (n = 3). Eighteen of these women died within 24 hours of admission, three died between 24 and 48 hours while one died on the 7th day after admission. Contributory factors to maternal death in women who died undelivered were identified as failure of the patient to seek appropriate medical care in time (n = 5), delay in referral from other healthcare facilities (n = 15) and delay in receiving care in our hospital due to administrative and patients' orientated factors (n = 18). Delay in instituting necessary care in our hospital were related to inability of the patients' relatives to adequately procure blood for transfusion for cases related to severe blood loss and anaemia, inability to pay for the required medical/surgical intervention, delay in accurate diagnosis by attending personnel and lack of appropriate facilities for management.

For those who delivered, mode of delivery was by spontaneous vertex delivery (n = 20), vaginal breech (n = 2), emergency caesarean section (n = 24), vacuum/forceps (n = 2), and destructive operation (n = 1). A total of 27 deaths were recorded among 545 women who had operative deliveries during the period of review compared to 22 deaths in the 2161 patients who had non-operative deliveries. Maternal death was therefore more likely to follow operative deliveries than non-operative deliveries (OR: 5.07; CI: 2.77–9.31). Review of the fetal outcome in those who delivered showed that there were 17 stillbirths and five early neonatal deaths. When considered with fetuses of women who died undelivered (that inevitably died), this translated to a perinatal death rate of 62.0%. Overall, 31 (41.3%) women died within 24 hours of hospital admission, while five (6.7%), 27 (36%) and 12 (16.0%) died within 24–48 hours, 2–5 days and >5 days of hospital stay respectively.

**Figure 1:**
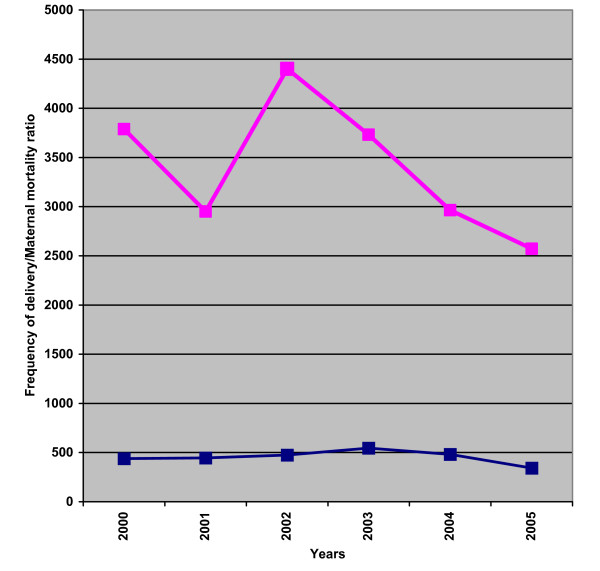
Trend in the frequency and delivery and maternal mortality ratio (2000–2005).  Deliveries.  Maternal mortality ratio (MMR).

The overall MMR for the reviewed period was 2989.2 per 100,000 live births. This figure is significantly higher than the 1,936.1 per 100,000 live births reported for 1988–1997 [8] in the same institution (75/2509 vs 103/5320, OR: 1.56, CI: 1.14–2.13; p = 0.0035). Table 3 shows the yearly trend of the number of deliveries and the corresponding MMR with their confidence limits. The MMR peaked in 2002 but had since dropped consistently (Figure 1). There was however no significant difference in the delivery rates and the MMR per year.

**Table 3:** Trend in the frequency of delivery and maternal mortality ratio (2000–2005)

**Year**	**Deliveries**	**Live births**	**Maternal deaths**	**MMR**	**(95% CI)**
2000	439	418	14	3349.3	1589.8 – 5108.8
2001	445	399	10	2506.3	940.7 – 4070.9
2002	475	433	17	3926.1	2059.6 – 5792.6
2003	545	502	16	3187.3	1620.1 – 4754.5
2004	481	443	11	2483.1	1005.3 – 3960.9
2005	343	314	7	2229.3	563.2 – 3895.4
Total	2728	2509	75	2989.2	563.2 – 3895.4

* Only the first half of 2005.

MMR: Maternal mortality ratio.

95% CI: 95% confidence interval

## Discussion

This study provides a baseline data on maternal mortality at the beginning of this millennium for a low resource tertiary care hospital in Nigeria as part of the local efforts to make MDG for maternal health a reality by 2015. The results show that maternal death remains a major public health issue not only in this hospital but also in Ogun State, Nigeria in view of the absolute number of deaths recorded in five and a half years. The MMR of 2989.2 per 100,000 live births in this series is significantly higher than the previously reported ratio in the same institution for 1988–1997 [8] but comparable to the figures recently quoted in other Nigerian tertiary centres [9,10]. This implies that the overall quality of maternal care in this hospital is poorer than in the previous decade and the present strategies to make pregnancy safer are yet to achieve the desired changes.

Though it appears reasonable to attribute the high MMR in this study to the low frequency of deliveries and hospital recruitment of cases, it is unlikely that this figure overestimates the true picture in the community. This is because 60% of deliveries in Nigeria take place outside the health facility and the outcomes of such deliveries are generally poorer than those managed in the hospital [11]. This observation is supported by the finding of a population-based study on maternal mortality in North-Central Nigeria, which showed a much higher MMR compared to hospital-based studies previously conducted in the same region [12]. Nevertheless, the proliferation of private hospitals and traditional birth attendant homes in Ogun State, which has consistently limited the total number of deliveries in our centre, could have biased our estimated mortality ratio more so that complicated cases are often referred from these facilities. It should be noted however, that the absolute number of maternal death is a self-evident measure that gives a clear indication of the public health impact of maternal mortality [13] and irrespective of the size of the denominator used for estimating our MMR, a total of 75 maternal deaths in such a short period is unacceptably high by any standard.

The study reiterates the importance of proper antenatal care and delivery towards reducing maternal mortality in this environment. Similar to the findings in previous studies in Nigeria [10,11], women unbooked for antenatal care and delivery at OOUTH, Sagamu were up to 22 times as likely to die in the hospital compared to booked patients. The percentage of booked patients who died probably reflects the likely MMR if all women were to have adequate antenatal care and well-supervised delivery. Though the contribution of unbooked emergencies to the present level of maternal death was not surprising, it is disturbing to note that a significant proportion of affected women had received some form of antenatal care and attempted to deliver at either a primary or secondary maternity unit within the State before their referral. The extent of complications in these women at presentation to our centre, as evident by the short period of admission before death, questions the quality of primary and secondary healthcare provided in our environment. It also causes serious concern as majority of the populace have better access to medical care at these levels of healthcare delivery. This observation, however, does not exonerate our institution since the incidence of maternal deaths among referred patients presenting to a referral centre is a reflection of its capability to handle emergencies. A situation where 8.4% of unbooked patients died suggests a deficiency in the provision of both basic and comprehensive emergency obstetric care in our obstetric unit.

The leading causes of maternal deaths in this study are not significantly different from those identified in the developing countries for several decades [14]. This implies that our pregnant women are still dying from preventable causes of maternal deaths and unlike suggested by some authors [15], no special technology or research is required to tackle the problem in this part of the world. It is interesting to note, however, that the contributions of the major causes of maternal death in this study vary slightly from that of earlier review in our centre. Eclampsia, the leading cause of maternal death in 2000–2005 (24.0%), was the third most common cause of maternal death in the earlier study (12.6%) while uterine rupture, which was responsible for only 8.0% of maternal deaths in 2000–2005, was the most common cause of maternal death in 1988–1997 (28.2%). Though this may reflect variation in the standard of care for these obstetric complications, other factors like the total number of such complications managed during the respective periods are required to arrive at such conclusion. In keeping with previous reports in Nigeria [8-10], pulmonary embolism is not an important cause of maternal deaths in this institution. Although diagnostic limitations may result in underreporting of this condition, our finding probably reflects the true picture of this complication in our community.

The large contribution of eclampsia to maternal deaths in this institution may be related to the existing management policy for hypertensive disorders in pregnancy. Up till now, magnesium sulphate, which has been shown in systematic reviews to reduce the risk of maternal mortality in eclamptics [16,17], is yet to be adopted for use in this institution due to non-availability. Therefore, efforts to reduce maternal deaths in this centre should include urgent adoption of a clear and up-to-date evidence-based protocol for the management of eclampsia. As expected, HIV-related maternal mortality is still quite low, probably reflecting the low prevalence of HIV-infection in this part of Nigeria [18]. However, any lapses in the strategies to limit the incidence of new HIV-infections in women might make this indirect cause of maternal death assume a larger proportion in the future, as is presently the case in some African countries [19].

Grandmultiparity is a well-recognised risk factor for maternal mortality in the tropics as shown by the extremely high MMR for this parity group in this study. Similar to the findings of Ogedengbe and Ogunmokun [20], the ratio was however not statistically different from that of nulliparae and Para 1–4 probably because most patients who died were women referred with complications from other facilities and were therefore not exhibiting the inherent obstetric risks associated with specific parity groups if labour were being managed by skilled attendants from onset.

A major limitation of this study is its retrospective nature, which precluded detailed understanding of the social and economic circumstances associated with maternal deaths in this hospital. Likewise, maternal deaths in the puerperium could have been underreported as postnatal clinic attendance in the hospital is generally poor and there is presently no measure to conduct home-based follow-up of parturients.

## Conclusion

In summary, ten years from the deadline of United Nations' MDG, pregnancy and childbirth remains very unsafe in Sagamu, Southwest Nigeria and the situation is worse than in the previous years. The present MMR suggests that access of women in Ogun State, Nigeria to proper antenatal care and delivery services is still poor and the quality of care provided in the State's referral hospital falls below expectation. It is apparent that the problems posed by referred pregnant women in this region is unlikely to disappear soon and certainly not before 2015. Therefore, this centre, as well as those in similar setting, should be poised for managing life-threatening complications in referred patients if the current trend of maternal deaths is to be kept in check. This can be achieved by updating the management policy for eclampsia and focussing attention on enhancement of the quality of emergency obstetric care particularly for the identified leading causes of maternal deaths. Improvement in blood banking and transfusion services and as well as provision of appropriate facilities for managing complicated cases would go a long way in achieving safer motherhood for women in this community.

## Competing interests

The author(s) declare that they have no competing interests.

## Authors' contributions

OT conceived and designed the study. OT and MA collected and analysed the data. OT drafted the manuscript while MA and TA revised it for intellectual contents.

## Pre-publication history

The pre-publication history for this paper can be accessed here:



## References

[B1] Haines A, Cassels A (2004). Can Millennium Development Goals be attained?. BMJ.

[B2] Graham W, Williams B, Snow R (1989). Estimating maternal mortality: the Sisterhood method. Stud Fam Plann.

[B3] AbouZahr C, Berer M, Ravindran TS (2000). Measuring maternal mortality: what do we know. Safe motherhood initiatives: critical issues.

[B4] Kampikaho A, Irwig LM (1991). Incidence and causes of maternal mortality in five Kampala hospitals, 1980–1986. East Afr Med J.

[B5] Geelhoed DW, Visser LE, Asare K, Schagen van Leeuwen JH, van Roosmalen J (2003). Trends in maternal mortality: a 13-year hospital-based study in rural Ghana. Eur J Obstet Gynecol Reprod Biol.

[B6] World Health Organization (1993). ICD-10: International statistical classification of diseases and health-related problems Tenth Revision.

[B7] Centers for Disease Control and Prevention (CDC), World Health Organization (2002). Epi Info 2002. Database and statistics software for public health professionals.

[B8] Sule-Odu AO (2000). Maternal deaths in Sagamu, Nigeria. Int J Gynecol Obstet.

[B9] Daramola AO, Banjo AA, Elesha SO (2004). Maternal deaths in the Lagos University Teaching Hospital: a ten-year review (1989 – 1998). Niger Postgrad Med J.

[B10] Uzoigwe SA, John CT (2004). Maternal mortality in the University of Port Harcourt Teaching Hospital, Port Harcourt in the last year before the new millennium. Niger J Med.

[B11] Onwudiegwu U, Ezechi OC (2001). Emergency obstetric admissions: late referrals, misdiagnoses and consequences. J Obstet Gynaecol.

[B12] Adamu YM, Salihu HM, Sathiakumar N, Alexander GR (2003). Maternal mortality in Northern Nigeria: a population-based study. Eur J Obstet Gynecol Reprod Biol.

[B13] Fortney JA (1987). The importance of family planning in reducing maternal mortality. Stud Fam Plann.

[B14] Harrison KA Maternal mortality in Nigeria. Paper commissioned by the United Nations Fund for Population Activities (UNFPA) for the International Conference of the Society of Gynaecology and Obstetrics of Nigeria at Abuja, September 13, 1990.

[B15] Tsu VD (2005). Appropriate technology to prevent maternal mortality: current research requirements. BJOG.

[B16] Duley L, Henderson-Smart D (2003). Magnesium sulphate versus diazepam for eclampsia. The Cochrane Database of Systematic Reviews.

[B17] Duley L, Gülmezoglu AM, Henderson-Smart DJ (2003). Magnesium sulphate and other anticonvulsants for women with pre-eclampsia. The Cochrane Database of Systematic Reviews.

[B18] Federal Ministry of Health Nigeria (FMOH) (2003). A technical report on the 2003 national HIV/Syphilis sentinel survey among pregnant women attending antenatal clinics in Nigeria.

[B19] Fawcus SR, Coeverden de Groot HA, Isaacs S (2005). A 50-year audit of maternal mortality in the Peninsula Maternal and Neonatal Service, Cape Town (1953–2002). BJOG.

[B20] Ogedengbe OK, Ogunmokun AA (2003). Grandmultiparity in Lagos, Nigeria. Niger Postgrad Med J.

